# GogB Is an Anti-Inflammatory Effector that Limits Tissue Damage during *Salmonella* Infection through Interaction with Human FBXO22 and Skp1

**DOI:** 10.1371/journal.ppat.1002773

**Published:** 2012-06-28

**Authors:** Ana Victoria C. Pilar, Sarah A. Reid-Yu, Colin A. Cooper, David T. Mulder, Brian K. Coombes

**Affiliations:** Michael G. DeGroote Institute for Infectious Disease Research and the Department of Biochemistry and Biomedical Sciences, McMaster University, Hamilton, Ontario, Canada; Stanford University, United States of America

## Abstract

Bacterial pathogens often manipulate host immune pathways to establish acute and chronic infection. Many Gram-negative bacteria do this by secreting effector proteins through a type III secretion system that alter the host response to the pathogen. In this study, we determined that the phage-encoded GogB effector protein in *Salmonella* targets the host SCF E3 type ubiquitin ligase through an interaction with Skp1 and the human F-box only 22 (FBXO22) protein. Domain mapping and functional knockdown studies indicated that GogB-containing bacteria inhibited IκB degradation and NFκB activation in macrophages, which required Skp1 and a eukaryotic-like F-box motif in the C-terminal domain of GogB. GogB-deficient *Salmonella* were unable to limit NFκB activation, which lead to increased proinflammatory responses in infected mice accompanied by extensive tissue damage and enhanced colonization in the gut during long-term chronic infections. We conclude that GogB is an anti-inflammatory effector that helps regulate inflammation-enhanced colonization by limiting tissue damage during infection.

## Author Summary

Bacterial pathogens have evolved sophisticated ways to subvert the innate defenses of their host. One way in which pathogens do so is by blocking or dampening the inflammatory response that is triggered once a microorganism is detected by the innate immune system. In this way, the microorganism can limit the activation of innate defenses in the host to promote its own colonization and dissemination. In this work we found that the enteric human pathogen *Salmonella enterica* serovar Typhimurium limits the activation of innate immune defenses in the host by using a bacterial protein called GogB to interfere with NFκB activation. NFκB is a key human transcription factor involved in the expression of pro-inflammatory cytokines during infection. In this infection situation, *Salmonella* delivers GogB to the infected cell where it interferes with ubiquitination of the NFκB inhibitor protein called IκBα to prevent translocation of NFκB to the nucleus where it would normally activate pro-inflammatory gene expression. The anti-inflammatory property of GogB is important for the bacteria to reach optimal infection densities in host tissues and to actively limit the tissue damage associated with prolonged inflammatory responses.

## Introduction

Horizontal gene transfer (HGT) is an important contributor to the genetic and phenotypic diversity of enteric bacteria. Plasmids and genetic regions called pathogenicity islands (PAI) can provide a fitness advantage and the ability to colonize and expand into novel host niches [1]. HGT has been a major driver of evolution of various *Salmonella enterica* serovars, giving rise to pathogens that infect a wide range of cold and warm-blooded animal hosts. *S. enterica* is a facultative intracellular pathogen that invades and replicates within host cells. *S. enterica* serovar Typhimurium (*S.* Typhimurium) causes food poisoning in humans characterized by diarrhea, abdominal pain and fever. The acquisition of two pathogenicity islands, *Salmonella* Pathogenicity Island-1 (SPI-1) and SPI-2, is considered a major factor in the evolution of *S.* Typhimurium pathogenesis. These genomic islands encode two different type III secretion systems (T3SS-1 and T3SS-2) that deliver effector proteins into host cells to manipulate host machinery leading to bacterial uptake, growth, and dissemination. T3SS-1 promotes invasion of epithelial cells, macrophage apoptosis and recruitment of phagocytes [2], [3] whereas T3SS-2 is primarily required for intracellular replication, dissemination, and disease associated with systemic spread [4]. SPI-1 and SPI-2 mutants are severely attenuated for virulence in mice infections [5] indicating that their concerted activities profoundly influence the host-pathogen interaction.

Acquisition of phage genes by lysogenic conversion contributed to the genetic diversity of *Salmonella* by providing an extended repertoire of virulence determinants that have integrated into ancestral regulatory networks of the bacterial cell [6]. GogB is a secreted effector encoded in the Gifsy-1 prophage found in some *S. enterica* strains and is a substrate of both T3SS-1 and T3SS-2 [7]. We showed previously that GogB is a chimeric protein consisting of an N-terminal canonical leucine-rich repeat domain (LRR) and a C-terminal domain with similarity to known proteins. *Salmonella* translocates GogB into the host cytoplasm, however its function and host cell target(s) were not known. The LRR domain in GogB resembles other LRR-containing effectors that function as a novel class of E3 type ubiquitin ligases called NELs [8], [9]. These include the *Salmonella* effectors SspH1, SspH2, and SlrP, and the IpaH family of proteins from *Shigella*.

The host ubiquitination system regulates a wide array of cellular processes including growth and differentiation, gene expression, and regulation of the host inflammatory response [10]. As such, host ubiquitination is a well-known target of bacterial as well as viral proteins [11], [12], [13], [14]. For example, the *Shigella* effector OspG binds to the Skp, Cullin, F-box containing complex (SCF) component UbcH5 to inhibit IκBα degradation and NFκB activation. In *Legionella*, interaction of the F-box protein AnkB with SCF ligase promotes intracellular replication within the host cell [12]. Viral pathogens among the *Poxviridae* family also target the SCF complex indicating a widespread mechanism of manipulating host cells during infection [14], [15]. As well, *Salmonella* exploits host ubiquitination pathways to regulate the temporal and spatial activity of SopE, SptP, and SopB effector proteins [16], [17], [18]. Other NEL effectors act as ubiquitin ligases for certain host cell targets [9].

The SCF complex is a multi-protein E3 ubiquitin ligase catalyzing the ubiquitination of proteins fated for degradation. The E3-type SCF^β-TrcP^ ligase complex regulates the NFκB pathway by targeting ubiquitinated IκBα for degradation by the proteasome [19]. The F-box domain is a ∼50 amino acid motif that mediates protein–protein interactions, often in concert with other protein-protein interaction platforms such as LRRs. The F-box motif interacts directly with the SCF protein Skp1 to bring specific protein targets to the E3 ligase [20]. Humans have at least 38 genes encoding different F-box proteins, although most of their functions and substrates are not known [21].

In this study, we identified the host cell target of the *Salmonella* effector GogB to be F-box only protein 22 (FBXO22). We mapped the FBXO22 interaction domain of GogB and showed that this interaction targets GogB to the SCF ubiquitin ligase complex to dampen the host inflammatory response by inhibiting IκBα degradation and NFκB activation. Consistent with this, a *gogB* mutant was hyper-inflammatory in the murine gut during chronic infection with an accompanying increase in tissue pathology and bacterial burden in gut tissue. We conclude that GogB is an anti-inflammatory effector that dampens host inflammatory responses following colonization in order to limit tissue damage and to balance bacterial colonization levels during chronic infection.

## Results

### GogB interacts with the SCF ubiquitin ligase

**Figure 1:**
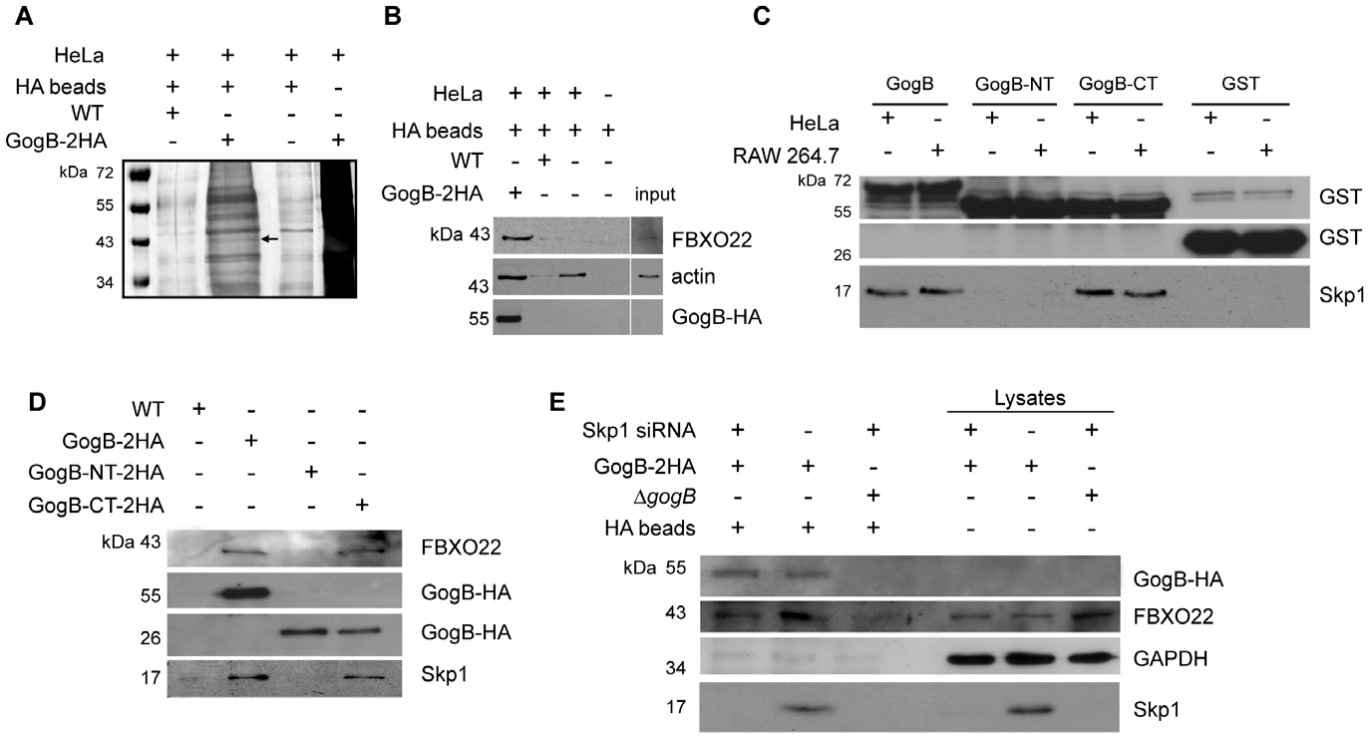
GogB interacts with FBXO22 and Skp1 of the SCF E3 ubiquitin ligase. **A.** Identification of GogB interacting partner in the host cell. GogB-HA delivered to HeLa cells by wild type *Salmonella* was immunoprecipitated and associated proteins were separated by SDS-PAGE. A silver-stained SDS-PAGE shows a ∼40 kDa band identified as F-box only protein (FBXO22) by mass spectrometry (*arrow*). **B.** Western blot analysis of the GogB-HA immunoprecipitation using antibodies against FBXO22 and actin. **C.** GogB interacts with the SCF ubiquitin ligase adapter protein Skp1 through the GogB C-terminus in a GST pull-down assay. GST-GogB and truncated GogB mutants encoding amino acid residues 1–253 (GogB-NT) and 254–497 (GogB-CT) were incubated with RAW 264.7 or HeLa cell lysates. Protein complexes were analyzed by Western blot using anti-Skp1 and anti-GST antibodies. GST beads alone mixed with lysates were used as control for non-specific binding. **D.** GogB immunoprecipitates a complex consisting of Skp1 and FBXO22 in host cells. GogB-HA or the truncation mutants were delivered to HeLa cells by the *Salmonella* T3SS and immunoprecipitated from infected cell lysates. Bound proteins were analyzed by Western blot using anti-Skp1 and FBXO22 antibodies. **E.** GogB interacts with FBXO22 in the absence of Skp1. HeLa cells were transfected with 80 pmol of either Skp1 siRNA or control siRNA. At 24 hr post-transfection, cells were infected with Δ*gogB* or Δ*gogB* complemented with pgogB-2HA. Cell lysates were mixed with anti-HA affinity beads after 20 hr post-infection and bound protein complexes were analyzed by Western blot using anti-Skp1, anti-FBXO22, and anti-HA antibodies. Anti-GAPDH was used as loading control for lysates.

Our previous work with GogB revealed an N-terminal LRR domain with similarity to the LRR-containing *Salmonella* effectors SspH1, SspH2, and SlrP and *Shigella* IpaH7.8/9 that have been characterized as NEL proteins [7], [8], [9]. The NEL domain has a conserved catalytic cysteine residue in the C-terminus that mediates E3 ubiquitin ligase activity *in vitro* [8], [22]. In order to identify potential host cell targets of GogB, we immunoprecipitated GogB-HA that had been delivered into HeLa cells by *Salmonella* via the T3SS (**Fig. 1A**). We identified three bands that co-purified only with GogB-HA and mass spectrometry analysis identified these proteins as lactate dehydrogenase, α-actin, and FBXO22, an uncharacterized ∼40 kDa human F-box-containing protein component of the SCF ubiquitin ligase complex. To determine whether the precipitated proteins specifically interacted with GogB, the pull-down assay was repeated and we confirmed that GogB specifically targeted FBXO22, whereas α-actin was a non-specific contaminant of the pull-down (**Fig. 1B**). F-box proteins function as the substrate recognition module of the E3-type SCF ubiquitin ligase complex by binding to the adapter protein Skp1 [23] thereby targeting substrates for degradation [21]. To explore the GogB-FBXO22 interaction further, we tested whether GogB also interacts with Skp1. A pull-down assay with GST-GogB mixed with lysates from either RAW264.7 or HeLa cells confirmed that Skp1 also co-purified with GogB (**Fig. 1C**). The Skp1 binding domain on GogB was identified by separately expressing the GogB N-terminal LRR domain (amino acids 1–253; *gogB-NT*) and the C-terminus encoding residues 254–497 (*gogB-CT*) and purifying each as GST fusions. GST pull-downs with these GogB fragments showed that Skp1 bound to GogB-CT but not to GogB-NT or GST alone (**Fig. 1C**). To verify this interaction in host cells, GogB-NT and GogB-CT (the C-terminal GogB fragment fused to the T3SS secretion signal [24]), were expressed as HA-tagged proteins in a Δ*gogB* mutant and verified to be secreted by the T3SS (Fig. S1). Similar to the GST pull-downs, GogB and GogB-CT but not GogB-NT delivered to host cells by the *Salmonella* T3SS interacted with a complex containing both Skp1 and FBXO22 as denoted by the presence of both proteins in lanes containing GogB and GogB-CT (**Fig. 1D**). Interestingly, we only observed FBXO22 co-purifying with GogB when delivered directly to host cells but not in GST pull downs with cell lysates indicating that targeting FBXO22 by GogB is specific to the infected host cell. These results demonstrate that GogB interacts with the SCF ubiquitin ligase complex and that the C-terminal domain of GogB mediates GogB-Skp1 and GogB-FBXO22 interactions. To determine whether Skp1 is required for the binding of GogB to FBXO22, we performed RNA interference experiments to knockdown the expression of Skp1 in HeLa cells. Lysates from infected HeLa cells shows that Skp1 expression was knocked down only in cells transfected with the Skp1 siRNA but not control siRNA. Infection of these transfected cells with Δ*gogB* complemented with the full-length GogB-2HA showed that FBXO22 was precipitated by GogB-2HA in the absence or presence of Skp1 (Fig. 1E). Pull-down assays were also performed using cells infected with Δ*gogB* as a control for non-specific binding. These results denote that GogB interacts with Skp1 and FBXO22 using different binding domains and that a GogB-FBXO22-Skp1 complex is formed in the host cells as shown by the presence of all three proteins in the pull-down assay using GogB-HA-infected cells. Whether FBXO22 is required for the binding of GogB to Skp1 remains to be determined as our attempts to knock down FBXO22 expression were not successful.

### GogB contains an Fbox-like motif at the C-terminal domain

**Figure 2:**
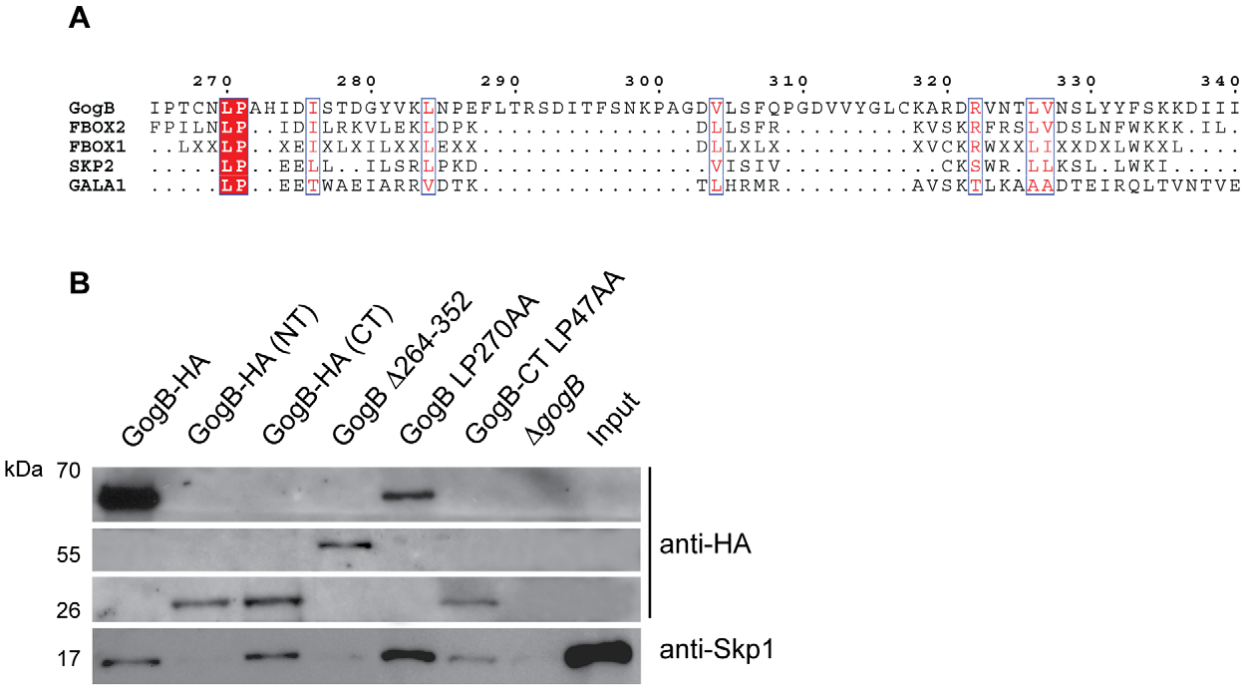
GogB contains an Fbox-like domain involved in Skp1 interaction. **A.** Amino acid sequence alignment of the putative Fbox-like domain found at the C-terminus of GogB with the F-box consensus sequences (FBOX1 and FBOX2), the human F-box protein Skp2, and the secreted effector GALA1 from the plant pathogen *Ralstonia solanacearum*. The conserved leucine and proline residues are highlighted in red and similar amino acid residues are highlighted in blue boxes. **B.** Deletion of the GogB Fbox-like domain abrogates binding to Skp1 *in vivo*. HeLa cells were infected with Δ*gogB* or Δ*gogB* expressing GogB, GogB-NT_1–253_, GogB-CT_254–497_, GogB-Δ264–352, a mutant with a deletion from residues 264–352, and the mutants GogB LP-AA and GogB-CT LP-AA in which the conserved leucine and proline residues in the Fbox-like domain were mutated to alanine. At 20 hr post-infection, infected cells were lysed and lysates were incubated with anti-HA affinity beads and bound proteins were resolved by SDS-PAGE and analyzed by Western blot using anti-Skp1 and anti-HA antibodies.

F-box proteins typically bind to Skp1 through a ∼50-amino acid F-box motif. We examined whether GogB also binds to Skp1 through a similar domain. Bioinformatic analysis of GogB identified an F-box-like domain between amino acids 270–334. Alignment of the GogB F-box-like motif with sequences from human F-box proteins β-Trcp1, Skp2, and the bacterial F-box secreted effector GALA1 from *Ralstonia solanacearum* [25] showed a conserved leucine-proline (LP) at positions 270–271 (**Fig. 2A**). Mutation of these residues in human Skp2 F-box abolished its interaction with Skp1 [26]. When we tested a GogB mutant (Δ264–352) lacking the F-box-like domain it was severely compromised in its ability to interact with Skp1 (**Fig. 2B**). Although the amounts of GogB-CT and GogB LP270AA bound to the HA-affinity beads were lower compared to that of full-length GogB (lane 1), this did not affect the interaction of these proteins with Skp1. In comparison, GogB-NT and GogB Δ264–352 bound to the affinity beads at similar amounts compared to GogB-CT or GogB LP270AA but these proteins did not precipitate Skp1, demonstrating that the binding of Skp1 is dependent on the F-box-like domain found at the C-terminus of GogB. To determine if the conserved leucine-proline residues in the GogB F-box-like domain were involved, we made alanine substitutions in full-length GogB and GogB-CT. Introducing L270A and P271A mutations in full-length GogB did not abolish binding to Skp1 (**Fig. 2B**). Although L47A and P48A mutations in GogB-CT appeared to reduce its interaction with Skp1, the amount of GogB-CT bound to the affinity beads is lower. We conclude from these results that GogB contains an F-box-like domain that is essential for Skp1 binding and that multiple determinants in this domain (perhaps including the conserved LP motif) are involved in this interaction.

### GogB interferes with IκBα degradation and NFκB activation

**Figure 3:**
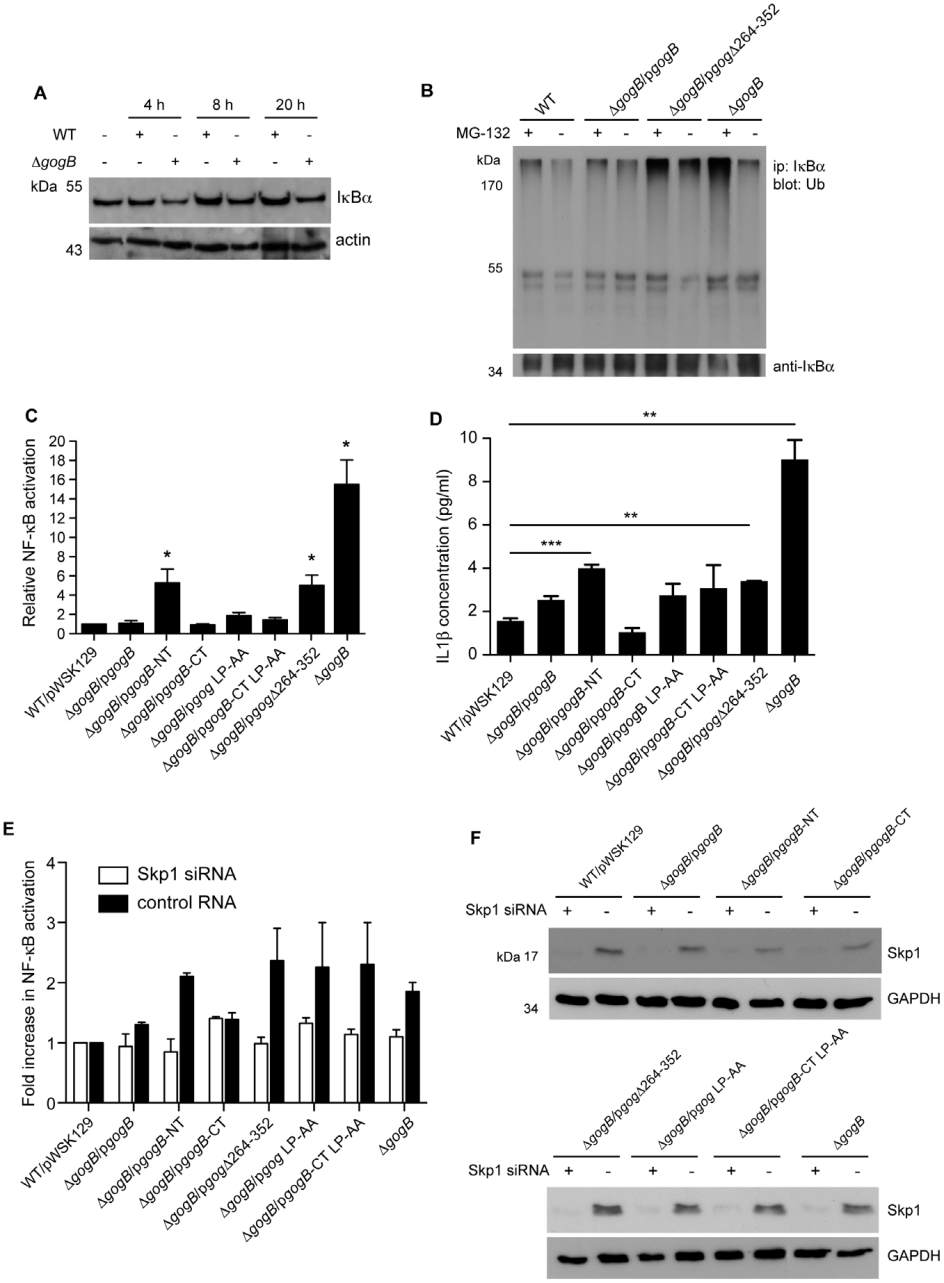
GogB decreases the host inflammatory response by inhibiting IκBα degradation and NFκB activation. **A.** GogB inhibits IκBα degradation during infection. RAW264.7 cells were infected with wild type and Δ*gogB Salmonella* and lysed after 4, 8, and 20 h. Lysates were probed using anti-IκBα antibody. Actin was used as a loading control. **B.** Deletion of GogB or the Fbox-like domain on GogB increases ubiquitination of IκBα. RAW 264.7 cells were infected with wild type *Salmonella*, Δ*gogB*, or Δ*gogB* complemented with plasmid encoding GogB or GogB-Δ264–352 in the presence (+) or absence (−) of the proteasome inhibitor MG-132. At 4 hr post-infection, cells were lysed and normalized by protein content. Levels of ubiquitinated IκBα were determined by a co-immunoprecipitation assay using IgA magnetic beads coupled to anti-IκBα antibodies and mixed with the lysates. Western blot was done using HRP-conjugated anti-ubiquitin (FK2) and rabbit anti-IκBα. **C.** GogB-deficient *Salmonella* induce higher NFκB activation compared to wild type. RAW264.7 were transfected with a luciferase reporter and then infected with wild type *Salmonella* containing an empty pWSK129 vector, Δ*gogB*, and Δ*gogB* strains complemented with plasmids encoding full-length GogB, GogB-NT_1–253_, GogB-CT_254–497_, GogB LP270AA, GogB-CT_254–497_ LP47AA, and GogB-Δ265–352. At 20 hr post-infection, NFκB-driving luciferase activity was measured and normalized to β-galactosidase levels and CFUs enumerated from each strain after infection. Data are expressed as mean fold difference in NFκB levels compared to cells infected with wild-type SL1344/pWSK129 with standard errors from three independent experiments. **D.** Deletion of GogB increases IL1β activation in infected macrophages. RAW264.7 cells were infected with wild type *Salmonella* containing an empty pWSK129 vector, Δ*gogB*, or Δ*gogB* complemented with plasmids encoding GogB, GogB-NT, GogB-CT, GogB LP270AA, GogB-CT_254–497_ LP47AA, and GogB-Δ264–352. At 20 hr post-infection, IL1β levels were measured in culture supernatants of infected macrophages by ELISA and normalized to CFUs enumerated from each strain at 20 h post-infection. Data are expressed as mean IL1β concentration (pg/mL) with standard errors from three independent experiments. Asterisks denote significant difference between the indicated strains, P<0.01. **E.** NFκB activation in Δ*gogB*-infected epithelial cells is blocked in the absence of Skp1. HeLa cells co-transfected with 2 pmol Skp1 siRNA or negative control siRNA, and a luciferase gene reporter system. At 24 hr post-transfection, cells were infected with wild-type *Salmonella*, Δ*gogB* or the complemented mutant strains. NFκB activation was determined by reporter gene assay after 20 hr post-infection. Values are normalized to β-galactosidase levels and CFUs enumerated from each strain after infection. Data are expressed as mean fold difference in NFκB levels compared to cells infected with wild-type *Salmonella* containing an empty vector. Assays were done in triplicate in three independent experiments (n = 3, ± SEM). **F.** Representative Western blot analysis showing knockdown of Skp1 expression in HeLa cells transfected with Skp1 or control siRNA and NFκB reporter assay and infected with wild-type SL1344/pWSK129, Δ*gogB* or Δ*gogB* complemented strains. Lysates were normalized by protein content and analyzed by Western blot using anti-Skp1 and anti-GAPDH as loading control.

The SCF^−βTRcP1^ complex is important for NFκB activation during the host inflammatory response because it targets the IκBα inhibitor protein for ubiquitination and degradation [19], [27]. Because other LRR-containing effector proteins are involved in modulating NFκB activity during infection, we investigated a potential role for GogB in the NFκB pathway through its interaction with the SCF ligase complex. To do so, we infected RAW264.7 cells with wild type and *gogB*-deficient *Salmonella* and monitored levels of IκBα. Western blot analyses showed that the amount of IκBα was lower in cell populations infected with a *gogB* mutant compared to wild type *Salmonella* (**Fig. 3A**). Non-virulent salmonellae have been shown to suppress the NFκB pathway by inhibiting ubiquitination of IκBα, thereby modulating the epithelial tissue response to inflammation [28]. To determine whether the GogB-dependent decrease in IκBα was due to ubiquitination and subsequent degradation, the level of poly-ubiquitinated IκBα was determined by infecting macrophages with wild type *Salmonella,* Δ*gogB*, and Δ*gogB* complemented with plasmids encoding GogB or GogB-Δ264–352 in the presence or absence of the proteasome inhibitor MG-132 to trap ubiquitinated IκBα molecules. In the absence of proteasome inhibition it was difficult to isolate ubiquitinated IκBα presumably due to its rapid degradation. However, in the presence of the proteasome inhibitor MG-132, cells infected with Δ*gogB* or bacteria expressing the non-functional GogBΔ264–352 mutant had higher levels of poly-ubiquitinated IκBα in IκBα pull-downs compared to the wild type strain. Complementation of Δ*gogB* with full-length GogB returned the levels of poly-ubiquitinated IκBα to that in wild type (**Fig. 3B**). These results suggested that GogB plays an essential role in down-regulating the host inflammatory response during *Salmonella* infection by inhibiting poly-ubiquitination of IκBα and thus NFκB-dependent gene expression. To test the latter, we measured NFκB-dependent expression of luciferase in RAW264.7 cells co-transfected with p*NFκB-luc* and pCMV*-βgal* reporter plasmids. Transfected cells were infected with wild type *Salmonella* containing an empty vector or the Δ*gogB* mutant and Δ*gogB* complemented with full-length *gogB*, or the mutant variants *gogB-NT*, *gogB-CT*, *gogB LP270AA*, *gogB-CT LP47AA*, and *gogB*Δ*264–352*. Macrophages infected with the *gogB* mutant had >10 fold higher luciferase levels compared to cells infected with wild type *Salmonella* (**Fig. 3C**). Deletion of the C-terminal domain previously shown to be the active fragment, or deletion of the F-box motif inhibited this NFκB-luciferase activity to ∼4-fold that of wild type. Complementation of Δ*gogB* with the full-length GogB or the Skp1-interacting domain, GogB-CT, resulted in NFκB-luciferase activity similar to that of wild type. However, mutation of the conserved leucine-proline residues in the F-box-like domain of GogB resulted in only a slight increase in levels of NFκB activity compared to wild type *Salmonella* denoting that other residues in the F-box motif may be needed to inhibit host inflammatory response, in keeping with our *in vitro* data with Skp1 interaction using the same GogB mutant. These results show that GogB partially blocks NFκB activation during *Salmonella* infection by inhibiting IκBα degradation through its interaction with Skp1 and that the GogB F-box-like domain is essential for this inhibitory activity. These data also support the idea that other regions of GogB, in addition to the F-box-like domain are involved in inhibiting NFκB activity.

### GogB inhibits IL1β production during infection

The functional relevance of the GogB-mediated decrease in NFκB activity during *Salmonella* infection was examined first by characterizing the inflammatory response of infected macrophages. RAW264.7 cells were infected with wild type *Salmonella*, Δ*gogB*, or complemented strains and interleukin-1β (IL1β) levels in culture supernatants were measured. Similar to the NFκB luciferase assay experiments, macrophages infected with wild type *Salmonella* or Δ*gogB* complemented with full-length GogB or GogB-CT secreted less IL1β compared to Δ*gogB* or the mutant complemented with GogB-NT or GogBΔFbox (**Fig. 3D**).

### Skp1 is necessary for modulation of NFκB by *Salmonella*

Previous work showed that the adapter protein Skp1 is essential for NFκB signaling [19]. To examine whether the increased activity of NFκB in cells infected with the *gogB* mutant was also dependent on Skp1 or whether it was signaled through an alternate pathway, we introduced the p*NFκB-luc* and pCMV*βgal* reporter plasmids into HeLa cells and then knocked down Skp1 expression by RNA interference (**Fig. 3E and 3F**). Thus, wild type *S.* Typhimurium and the *gogB* mutants are unable to activate NFκB in the absence of Skp1.

Luciferase assays showed that knockdown of Skp1 expression in host cells resulted in similar levels of NFκB activity upon infection of cells with Δ*gogB* and complemented strains compared to the wild-type SL1344/pWSK129. In contrast, treatment of HeLa cells with control siRNA showed a 2 to 3 fold increase in NFκB activity in cells infected with Δ*gogB* and strains complemented with *gogB-NT* or strains with mutations in the F-box motif, gogB Δ*264–352, gogB LP270AA* and *gogB-CT* LP47AA (**Fig. 3E**). Similar to the luciferase assays performed using macrophages, Δ*gogB* complemented with the full-length GogB or GogB-CT had similar NFκB activity as wild-type cells containing an empty plasmid. Interestingly, HeLa cells infected with GogB mutants complemented with *gogB LP270AA* and *gogB-CT* LP47AA showed a higher NFκB level compared to wild-type *Salmonella*. These results show that differences in NFκB activation in cells infected with wild-type *Salmonella* and Δ*gogB* are abolished in the absence of Skp1. Overall, our results demonstrate that GogB plays a role in blocking the host inflammatory response through inhibition of NFκB activation, IκBα ubiquitination and degradation and that this modulation requires the interaction of GogB with Skp1 of the SCF ubiquitin ligase.

### GogB does not affect *Salmonella* replication in macrophages or during acute infection of susceptible mice

**Figure 4:**
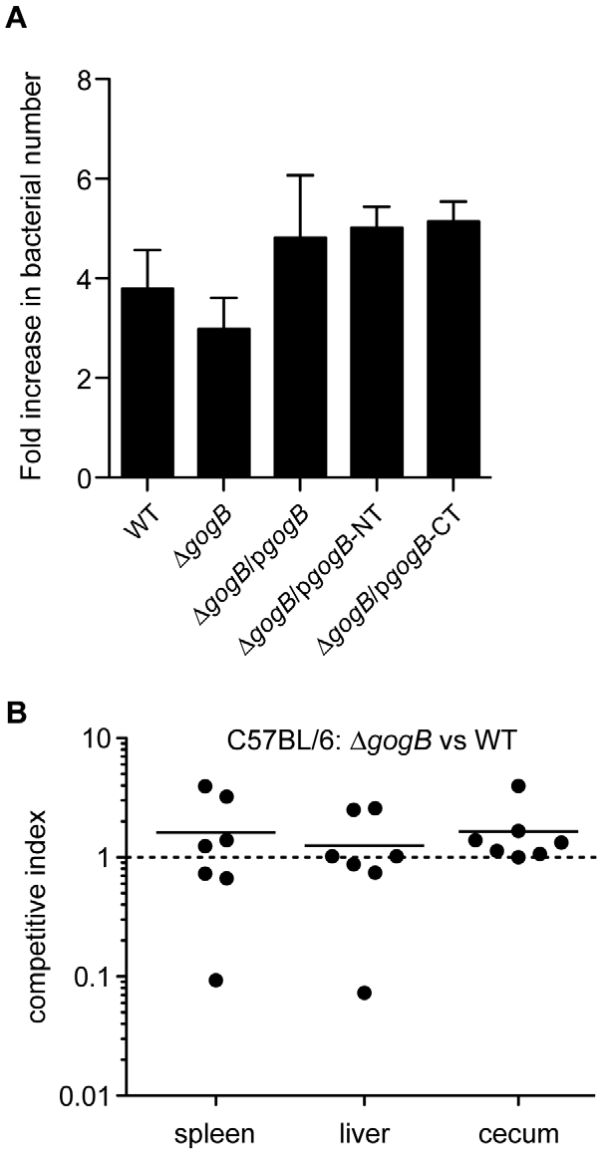
Deletion of GogB does not affect replication in cells *in vitro* or acute infection of susceptible mice. **A.** RAW 264.7 cells were infected with wild type *Salmonella*, Δ*gogB*, *ΔgogB*/p*gogB-2HA*, Δ*gogB*/*pgogBNT-2HA* and Δ*gogB*/*pgogBCT-2HA*. At 20 hr post-infection, infected cells were lysed and intracellular bacteria were enumerated. Fold replication represents the ratio of intracellular bacteria at 20 h and 2 h. Data are the means with standard error of three separate experiments. **B.** C57BL/6 mice were orally infected with a mixed inoculum of wild type *Salmonella* and the Δ*gogB* strain. The competitive index (CI) was determined in the spleen, liver and cecum after 3 days. Each data point represents one animal and horizontal bars indicate geometric means.

Our results demonstrating the role of GogB in modulating the host immune response through its interaction with the SCF ligase led us to examine the role of this effector during acute and chronic animal infections. We first performed gentamicin protection assays to determine whether deletion of *gogB* impaired *Salmonella* replication in macrophages and epithelial cells, which it did not, as invasion at 2 h post-infection and replication after 20 h were similar between the strains with and without GogB (**Fig. 4A**). This suggested that the role of GogB might manifest in the context of animal infection. To test this we used competitive acute infections of susceptible C57BL/6 mice infected with a mixture of wild type and Δ*gogB Salmonella*. Mutant and wild type bacteria were recovered in equal numbers from gut tissues and systemic organs after three days (**Fig. 4B**) indicating that deletion of *gogB* does not significantly affect *Salmonella* fitness over acute infection periods in genetically susceptible mice.

### GogB influences colonization dynamics and modulates inflammatory responses during infection of genetically resistant mice

**Figure 5:**
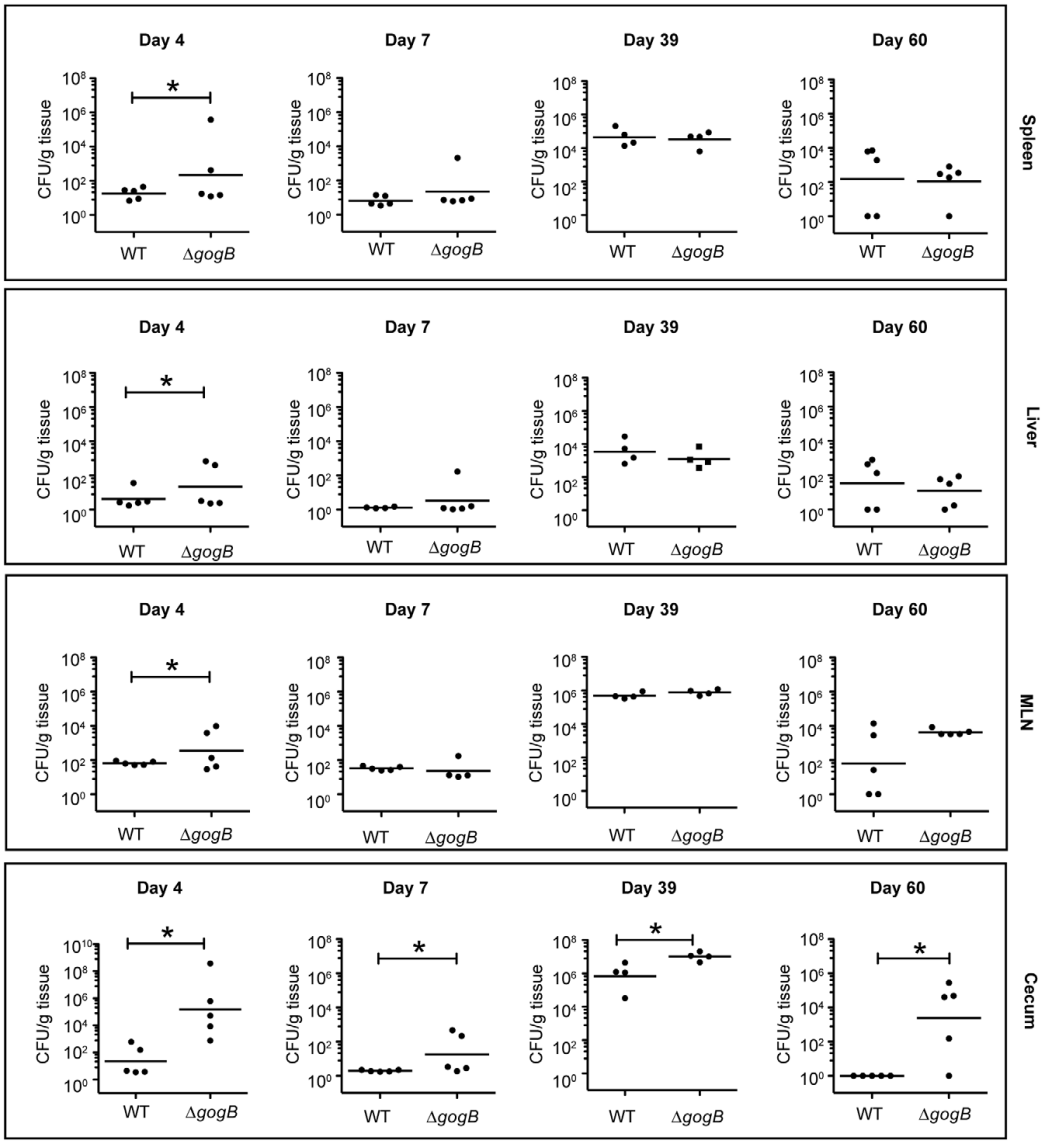
Deletion of GogB promotes chronic colonization in Nramp^+/+^ mice. Groups of 129/svImJ mice were orally infected with wild type *Salmonella* or the Δ*gogB* mutant. After 4, 7, 39, or 60 days of infection, viable bacteria were recovered from organs and enumerated. Each data point represents one animal and horizontal bars indicate geometric means. Asterisks denote significant differences between wild type and Δ*gogB* (P<0.05).

The Natural resistance-associated macrophage protein (Nramp)-1 controls intracellular *Salmonella* growth by limiting the transport of divalent cations across the bacterial vacuole [29]. Given that *gogB*-deficient *Salmonella* were not attenuated for acute infection of susceptible mice we tested whether the GogB-mediated modulation of the host inflammatory response contributed to chronic infection. We infected groups of Nramp1^+/+^ mice (129S1/SvImJ) with wild type or Δ*gogB Salmonella* and measured bacterial colonization in systemic and intestinal sites at 4, 7, 39, and 60 days after infection (**Fig. 5**). After four days, both wild type and Δ*gogB* colonized the mice, however, mice infected with *gogB*-deficient *Salmonella* had moderate but significantly higher bacterial load in all tissues (*p*<0.05), with the largest effect seen in the cecum. The Δ*gogB*-infected mice had enlarged mesenteric lymph nodes (MLN) yet we did not observe outward signs of acute illness such as hunching, ruffled fur or piloerection (data not shown). At time points greater than 7 days there was no difference between the number of wild type and mutant bacteria recovered from the spleen or liver. However, what was striking was the high degree of cecal colonization in mice infected with *gogB*-deficient *Salmonella* throughout the long-term infection coupled with greater bacterial load in the MLNs at day 60 (**Fig. 5**). Although the bacterial load differences in the MLN at day 60 did not reach statistical significance, all the Δ*gogB*-infected mice displayed a similar high degree of colonization whereas colonization by wild type *Salmonella* was more variable. From days 21 to 49, resistant mice are able to clear *Salmonella* or eventually progress to a chronic carrier state [30]. Indeed at day 60, very low numbers of wild type bacteria were recovered from the cecum whereas Δ*gogB* mutants were present at up to 5–6 log greater numbers, demonstrating a profound difference between wild type and Δ*gogB* to persist in the mouse cecum (**Fig. 5**). To gain preliminary insight into the connection between bacterial load and inflammation, we treated mice with dexamethasone as an immunosuppressant prior to infection with *Salmonella* and monitored bacterial load in the cecum at day 4 after infection (**Fig. S2**). In these experiments, the bacterial load of the *gogB* mutant was drastically reduced in immunosuppressed mice and similar to the levels of wild-type bacteria in the cecum during dexamethasone treatment. These data suggested to us that the *gogB* mutant does not have an inherent increased growth rate leading to increased inflammation, but rather benefits from a heightened inflammatory response that is suppressible by chemical blockade.

**Figure 6:**
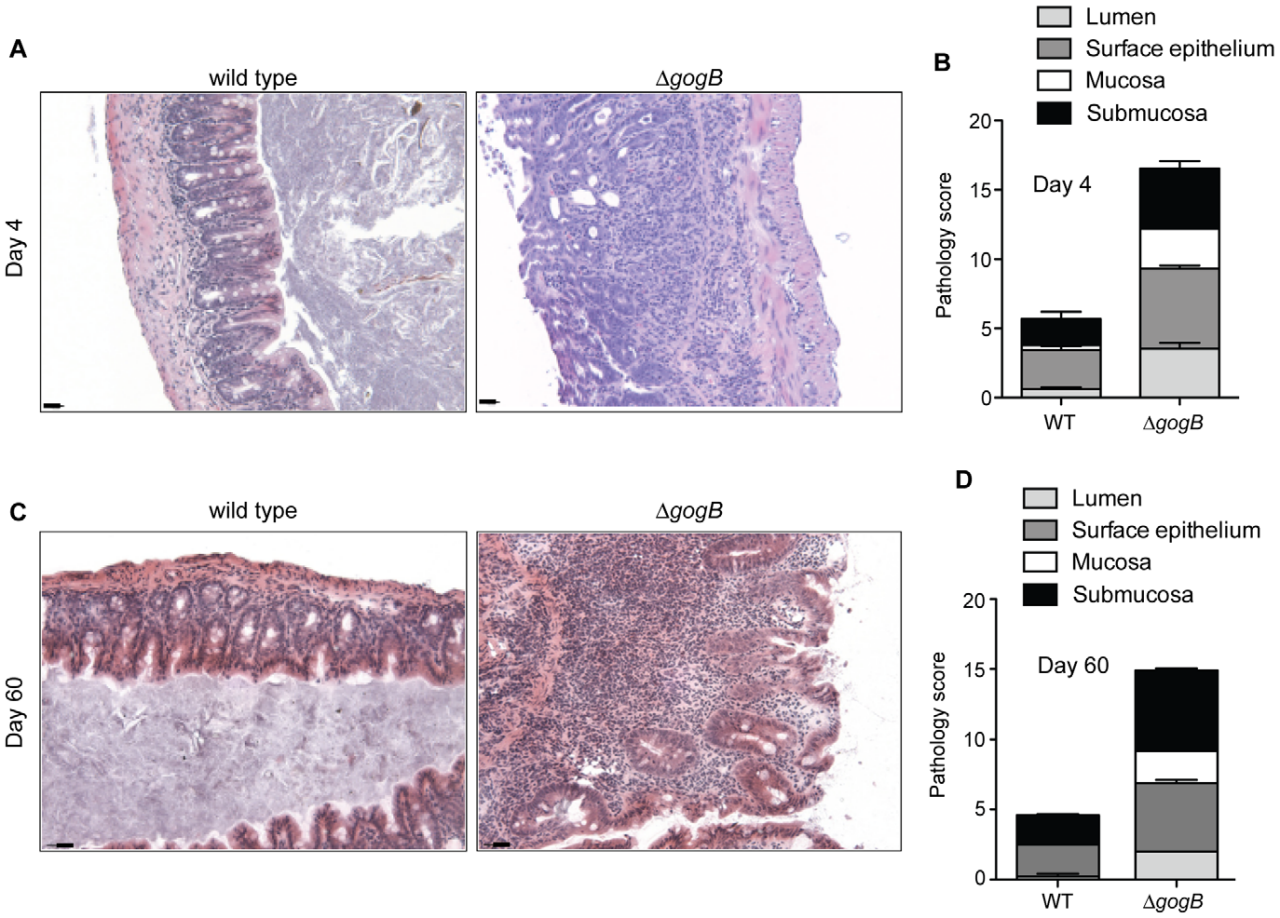
Cecal colonization in Δ*gogB*-infected mice is accompanied by inflammation and tissue damage. **A.** Hemotoxylin-eosin-stained cecal tissue from infected 129/svImJ mice at day 4. Images are representative of tissue from 5 mice per group, scored for pathology (B) as outlined in Methods. C. Hemotoxylin-eosin-stained cecal tissue from infected 129/svImJ mice at day 60. **D.** Tissue pathology at day 60 post-infection was quantified as described.

Histopathological analysis revealed a marked difference in tissue inflammation and integrity of the cecal tissue in mice infected with wild type and Δ*gogB* at day 4 (**Fig. 6A and 6B**) and at day 60 (**Fig. 6C and 6D**). Tissue samples from wild type-infected mice were characterized by mild to moderate infiltration of mononuclear cells and polymorphonuclear leukocytes (PMN) particularly at the surface epithelium and submucosa, minimal necrosis and edema, and minimal pathological changes in the mucosa. In comparison, increased colonization by Δ*gogB* was accompanied by moderate to severe pathology with dramatic changes in the gross morphology of the cecum due to increased influx of mononuclear cells and PMNs, granulomatous lesions, ulceration and increased frequency of cryptic abscesses.

**Figure 7:**
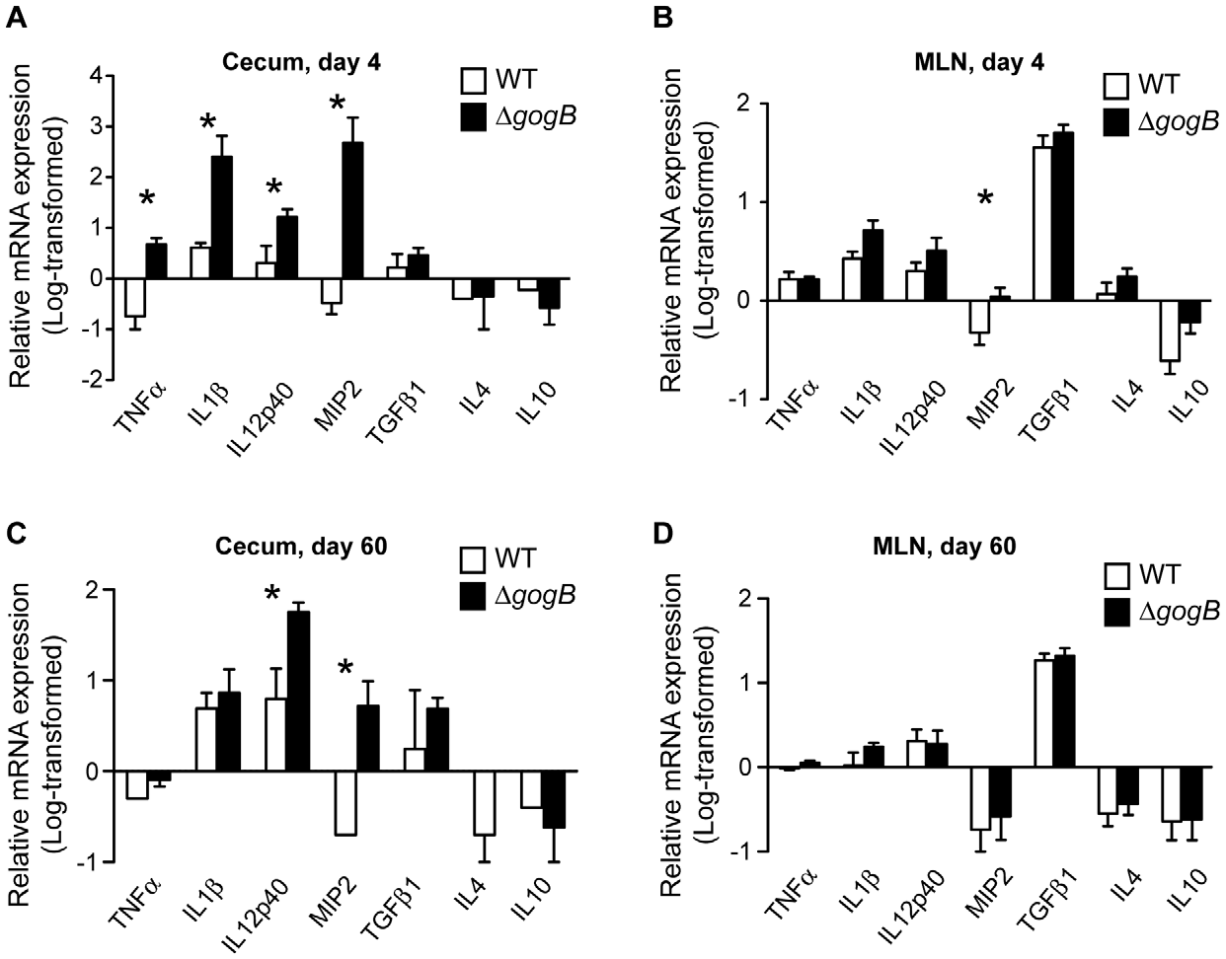
Expression of inflammatory markers in the cecum and MLN of infected mice. 129svImJ mice were orally infected with wild type or Δ*gogB Salmonella*. At day 4 post-infection (**A and B**) and day 60 post-infection (**C and D**), RNA was extracted from the cecum and MLN and the relative mRNA levels of the inflammatory markers shown were determined by RT-PCR. Relative expression levels were normalized to 18S RNA and expressed as log-transformed mean fold change in expression with standard errors from 5 experiments. Asterisks denote significant difference from mice infected with wild type and Δ*gogB Salmonella* (P<0.05).

From recent work, intestinal inflammation provides a selective advantage for *Salmonella* by creating an environment that enhances the pathogen's growth and dissemination via metabolic fitness [31], [32]. To examine the inflammatory markers associated with colonization and pathology, we measured the levels of pro- and anti-inflammatory cytokines in the cecum and MLN by quantitative RT-PCR. At day 4, expression of the NFκB target genes, TNFα, IL1β and IL12p40 were significantly upregulated in the cecum of Δ*gogB*-infected mice compared to wild type, while expression of TGFβ1 and the anti-inflammatory cytokines IL4 and IL10 were similar in both groups of mice (**Fig. 7A**). The expression profiles of pro- and anti-inflammatory cytokines in the MLNs were similar in both groups of infected mice except for MIP2 (**Fig. 7B**). NFκB activity is repressed by TGFβ1-mediated stabilization of IκBα [33] and the expression of TGFβ1 in the MLN from both groups of mice corresponded with an overall lower expression of NFκB target genes compared to the cecum. The higher expression of macrophage inflammatory protein 2 (MIP-2) chemokine in the cecum and MLN of Δ*gogB*-infected mice is consistent with the increased recruitment of phagocytes. At day 60, the levels of IL12p40 and MIP2 remained higher in the cecum of Δ*gogB*-infected mice (**Fig. 7C**) whereas the other inflammatory markers had normalized in the MLN between both groups of mice. Overall, these data indicate that GogB plays an important role in dampening the host immune response and limiting tissue destruction during *Salmonella* infection in the gut.

## Discussion

In this study, we characterized the role of the LRR-containing T3SS effector GogB in *Salmonella* pathogenesis. Due to its similarity in the LRR domain with members of the NELs, we hypothesized that GogB may be involved in subverting the host ubiquitination process. We identified FBXO22 as the host cell target of GogB that facilitates binding to host Skp1. Skp1 is an adapter protein linking diverse F-box proteins and Cullin-1 to form the SCF ligase complex [23]. Accordingly, we found that GogB interacted with Skp1, which was mediated by an Fbox-like domain in the C-terminal part of GogB. Thus, GogB could be functioning in a similar manner to the *Legionella* effector AnkB (Lpp2082) previously identified as a prokaryotic F-box protein and to the ankyrin repeat proteins found in poxvirus that contains an F-box motif at the C-terminal domain [12], [13], [14]. Whether or not the N-terminal LRR in GogB interacts with other host cell proteins or forms a complex with other effectors through this domain has yet to be determined, although we did show that this LRR domain is not necessary for targeting GogB to either Skp1 or FBXO22, or for the downstream inhibition of NFκB activation. Recently, one target of the SCF^FBXO22^ complex was identified as the histone demethylaase KDM4A [34]. However, another study showed that KDM4A turnover was instead coordinated by an SCF^FbxL4^ complex [35]. Therefore, whether or not GogB is involved in KDM4A turnover remains an open question that could be the focus of follow up work. To our knowledge, our report is the first to implicate a role for FBXO22 in the regulation of the NFκB pathway, thus broadening the function of this newly described human F-box protein.

In the human F-box protein Skp2, the last 30 amino acids of the LRR domain fold over the F-box-Skp1 interface, suggesting a possible mechanism by which the GogB LRR may be performing a regulatory function in the full-length protein [26]. The Skp2 F-box has three helices, with H1 folding orthogonally to the H2-H3 anti-parallel helices that interdigitate with the C-terminus of Skp1 [26]. Structure predictions for GogB show its F-box domain to be a combination of coiled-coils and helices. Given that the GogB F-box was necessary for Skp1 interaction, it seems this motif may perform a similar function to the Skp2 F-box in binding to Skp1. In the case of FBXO22 and GogB, it is possible that both proteins bind to Skp1 but the presence of GogB may prevent proper function of the SCF^FBXO22^ ligase, perhaps in conjunction with another protein(s). On this point, some of our data is consistent with the GogB-FBXO22 interaction being dependent on another molecule since FBXO22 co-purified with GogB only when this effector was delivered into host cells by bacteria and not when GogB was mixed with host cell lysates. This might implicate another bacterial-delivered effector or host protein in the interaction of GogB with FBXO22. Alternatively, it is increasingly being shown that host-dependent modification of bacterial effectors can dictate their biological function [36], [37]. As such, the GogB-FBXO22 interaction may be enhanced or stabilized by a host-dependent modification process following translocation. These possibilities will be the subject of work to follow.

Gut inflammation is important for colonization by *Salmonella* [31], [32]. A typical *Salmonella* infection elicits transient inflammation during the early stages of infection but does not lead to destruction of the intestinal epithelium [38]. The ability to elicit intestinal inflammation is dependent, in part, on both T3SS encoded in *Salmonella* and their associated effectors [39], [40], [41]. The SPI-1 T3SS is better known for its role in regulating the early colonization of the intestine, whereas the SPI-2 T3SS has typically been studied in the context of systemic infection. Although the role of these two T3SS in distinct stages of infection may be less disparate than previously thought, it is interesting that GogB is one of only a few effector substrates that is delivered by both T3SS-1 and T3SS-2 systems in *Salmonella* [7] indicating that this protein might be involved in regulating immune processes from an early stage of the host-pathogen interaction.

A major finding of biological significance in this study was that the GogB-mediated down-regulation of the host inflammatory response during chronic mouse infection limits tissue damage and host pathology. In the absence of GogB, mice were colonized to greater levels especially in the chronically infected cecum accompanied by increased inflammation and tissue damage. *Salmonella* reside within macrophages in the MLN, which serves as a reservoir for subsequent re-seeding of the liver and spleen [42], [43]. Our data on the levels of pro-inflammatory cytokine expression at days 4 and 60 post-infection showed that inflammation was prominent only in the cecum and not in the MLN. This might indicate that a subpopulation of Δ*gogB Salmonella* that are able to traverse the epithelial barrier and reach the MLN, liver and spleen retain their ability to suppress systemic host inflammation possibly through the concerted action of other anti-inflammatory effectors. The increased inflammation in gut tissue elicited by bacteria lacking GogB along with higher cecal colonization in Nramp1-positive mice by the *gogB* mutant supports recent data showing that *Salmonella* takes advantage of intestinal inflammation for metabolic fitness by using tetrathionate as a respiratory electron acceptor [31]. What was unclear however was how the pathogen might temper this host inflammatory response once infection is established to limit the tissue damage that arises from an unchecked immune response. The acquisition of the type III effector GogB with anti-inflammatory activity appears to benefit *Salmonella* by limiting host tissue damage during of colonization, while maintaining a degree of inflammation that supports competition with the host microbiota. In this way, GogB appears to balance the pathogen's short-term need for inflammation-enhanced colonization while moderating this immune response to limit tissue damage in the longer term.

## Materials and Methods

### Ethics statement

All experiments with animals were conducted according to guidelines set by the Canadian Council on Animal Care. The local animal ethics committee, the Animal Review Ethics Board at McMaster University, approved all protocols developed for this work.

### Bacterial strains and cell culture

All bacterial strains used in this study are listed in Table S1. *S. enterica* serovar Typhimurium strain SL1344 was used in this study and all mutant strains were derivatives of *Salmonella* SL1344. The generation of the *gogB* mutant was described previously [7]. *Salmonella* was grown in Luria-Bertani (LB) broth or on agar plates with appropriate antibiotics. *E. coli* strain BL21 (DE3) was used for expression and purification of recombinant GST-tagged GogB and truncated mutants. RAW264.7 and HeLa cells were routinely cultured in Dulbecco's Modified Eagle Medium (DMEM) supplemented with 10% fetal bovine serum (FBS) and grown at 37°C and 5% CO_2_.

### Plasmid construction

The plasmids and primers used in this study are listed in Table S1. The generation of the plasmid pWSK129-*gogB*-*2HA* encoding a hemagglutinin (HA)-tagged *gogB* that is expressed under its native promoter was described previously [7]. To generate the epitope-tagged mutant *gogB-NT_1–253_*, the region encoding amino acid residues 1 to 253 of *gogB* was PCR amplified using the plasmid pWSK129-*gogB-2H*A as a template and the product was digested with *Sal*I and *Bgl*II and cloned into the corresponding sites of pWSK129-2HA in which the *gogB* coding region was removed. Strand-overlap PCR was used to construct the HA-tagged mutant *gogB-CT_254–497_*. The *gogB* native promoter and the secretion signal sequence encoding the first 29 amino acids at the N-terminus were amplified by PCR. A second PCR was performed to amplify the *gogB* region encoding the C-terminus from amino acid residues 254 to 497. The resulting products were fused together by a third round of PCR, digested with *Sal*I and *Bgl*II and cloned into the corresponding sites of pWSK129-2HA. The conserved leucine (L270) and proline (P271) in GogB, and L47 and P48 in GogB-CT_254–497_ were substituted with alanine residues using the QuikChange site-directed mutagenesis kit (Stratagene) and the plasmids pWSK129 *gogB-2HA* and pWSK129 *gogBCT254–497-2HA* as templates. In-frame deletion of the identified F-box motif in GogB was done by strand-overlap PCR in which the upstream *gogB* promoter and the region encoding amino acid residues 1 to 264 were amplified. A separate PCR was performed to amplify the region spanning amino acid residues 353 to 497. The resulting products were fused together by PCR, digested with *Sal*I and *Bgl*II and cloned into the corresponding sites of pWSK129-2HA. The plasmids encoding epitope-tagged GogB and GogB mutants were transformed into Δ*gogB*. An in-frame marked deletion of *gogB* and *sspH2 or sseL* to produce a double gene knockout strain was performed by lambda red recombination. To generate GST-tagged *gogB* and the truncated mutants *gogBNT_1–253_* and *gogBCT_254–497_*, the corresponding regions were amplified by PCR using the pWSK129 templates described above (Table S1). The PCR products were digested with *Sal*I and *BamH*I and cloned into the corresponding sites of the plasmid pGEX-6P-1.

### Gentamicin protection assays

RAW264.7 or HeLa were seeded into 24-well plates at 5×10^5^ cells/ml 18 hr prior to infection. Overnight bacterial cultures were opsonized with 20% normal human serum and used to infect macrophages at a multiplicity of infection (MOI) of 10 or 20 bacteria per cell. To prepare highly invasive bacteria for infection of HeLa cells, overnight cultures were subcultured in LB and grown for 2 hr at 37°C with shaking. Bacteria were washed with PBS and resuspended in DMEM/FBS and used to infect epithelial cells at MOI of 10 or 20 bacteria per cell. After 30 min of infection, the media was replaced with DMEM/FBS containing 100 µg/ml gentamicin to kill extracellular bacteria and incubated for 1.5 hr. At 2 hr post-infection (PI), cells were lysed with PBS/1% Triton X-100 to release intracellular bacteria and the number of colony-forming units (CFU) was determined by plating serially-diluted bacteria on LB agar plates supplemented with 50 µg/ml streptomycin. Infected cells were washed with PBS and incubated with media containing 10 µg/ml gentamicin for longer time points. Bacterial numbers were expressed as the ratio of intracellular CFU at 20 h to 2 h.

### Protein purification

The procedure for purification and solubilization of recombinant GogB, GogB-NT and GogB-CT GST fusion proteins was performed as described previously with several modifications [44]. *E. coli* BL21 (DE3) were transformed with plasmids encoding GST alone or GST fusion proteins and grown in 1 liter LB with 100 µg/ml ampicillin to OD_600_∼0.7 at 37°C. Protein expression was induced with 0.1 mM isopropyl β-D-1-thiogalactopyranoside (IPTG) for 24 hr at 16°C. Cells were harvested, resuspended in lysis buffer containing 40 mM Tris pH 8.0, 500 mM NaCl, 1 mM EDTA, 50 µg/ml lysozyme and protease inhibitor cocktail (Roche). DTT was added to a final concentration of 5 mM then cells were lysed by sonication. A final concentration of 1% Triton X-100 was added to the lysate and incubated on ice for 20 min. The lysates were clarified by centrifugation and the supernatant was mixed with glutathione sepharose 4B matrix beads (GE Healthcare) previously equilibrated with 40 mM Tris pH 8.0, 500 mM NaCl (TBS). The mixture was incubated overnight at 4°C in a rotator-shaker then the column was washed with ten bed volume of TBS. Proteins were eluted by incubating beads overnight with elution buffer (40 mM Tris pH 8.0, 500 mM NaCl, 1% Triton X-100, 5 mM DTT and 50 mM reduced glutathione) at 4°C. Proteins were dialyzed against TBS with 1% Triton X-100, 5 mM DTT to remove excess glutathione and concentrated using an Amicon centrifugal filter (Millipore). Alternatively, GST beads coupled to recombinant proteins were washed with TBS and stored at 4°C in storage buffer (40 mM Tris pH 7.5, 500 mM NaCl, 1% Triton X-100, 5 mM DTT, 10% glycerol) until further use in GST pull-down assays.

### GST pull-down assays and *in vivo* co-immunoprecipitation

Purified recombinant GST-tagged GogB, GogB-NT, GogB-CT or GST were coupled to GST affinity beads. HeLa or RAW264.7 cells were lysed with lysis buffer containing 40 mM Tris pH 8, 200 mM NaCl, 1% Triton X-100 and protease inhibitor cocktail (TBST). Lysates were clarified by centrifugation and the supernatant was incubated with GST affinity beads overnight at 4°C with end-on-end mixing. The beads were washed under stringent conditions and resuspended in SDS sample buffer. Bound proteins were resolved by SDS-PAGE and analyzed by Western blot. Cell-based co-immunoprecipitation assays were performed by infecting HeLa or RAW264.7 cells with wild type SL1344, Δ*gogB* or a *gogB* mutant strain containing a plasmid that encodes HA-tagged *gogB* or *gogB* mutants. At 20 h post-infection, cells were washed with PBS and lysed with TBST. Lysates were incubated with anti-HA affinity beads (Roche) overnight at 4°C with end-on-end mixing. The beads were washed under stringent conditions and resuspended in SDS sample buffer. Bound proteins were resolved by SDS-PAGE and analyzed by Western blot.

### Luciferase reporter assays

RAW264.7 or HeLa cells were seeded into 96-well plates and after 16 h, cells were transfected with the reporter plasmid pNFκB-*luc* and the control plasmid pCMVβgal (Clontech) using Fugene HD reagent (Roche) according to manufacturer's recommendations. Twenty-four hours after transfection, cells were infected as described above. At 20 h post-infection, luciferase activity was measured using the Luc-Screen System (Applied Biosystems) and β-galactosidase levels were measured using Galacto-star system following the manufacturer's protocols. Luciferase signals were normalized to β-galactosidase levels and to colony forming units (CFUs) enumerated for each strain at 20 hr post-infection. Data were expressed as fold activation relative to cells infected with wild-type *Salmonella* containing an empty plasmid (mean ± SEM, *n*≥3).

### Skp1 knockdown in HeLa cells

The short-interfering RNA (siRNA) sequence used to knockdown Skp1 expression was published previously [13]. HeLa cells were seeded into 96 well plates and after 16 h, cells were co-transfected with 2 pmol of the Skp1 siRNA (GCA AGU CAA UUG UAU AGC AGA A and UUC UGC UAA UAC AAU UGA CUU GC), the reporter plasmid pNFκB-*luc* and the control plasmid pCMVβgal using Lipofectamine 2000 transfection reagent (Invitrogen). For control experiments, HeLa cells were co-transfected with a negative control siRNA and the reporter plasmids. At 24 h post-transfection, cells were infected at an MOI of 20 and the luciferase activity was measured as described above. Western blot analysis of HeLa cell lysates was performed to confirm knockdown of Skp1 expression using anti-Skp1 antibody and anti-GAPDH as loading control. For co-immunoprecipitation assays, HeLa cells were seeded into 24 well plates and after 16 h, cells were transfected with 80 pmol Skp1 or control siRNA using Lipofectamine 2000 reagent for 24 hr. Cells were then infected with either Δ*gogB* or Δ*gogB* complemented with pgogB-2HA at an MOI of 20. At 20 hr post-infection, cells were lysed and mixed with anti-HA affinity beads overnight at 4°C. Bound protein complexes were resolved by SDS-PAGE and analyzed by Western blot using rabbit anti-Skp1, mouse anti-FBXO22, and HRP-conjugated rat anti-HA. Lysates from infected cells were normalized by protein content and analyzed by Western blot to determine knockdown of Skp1 expression. Mouse anti-GAPDH antibodies were used as loading control.

### Measurement of IL1β

RAW264.7 cells were seeded in 96 well plates and infected as described above. At 20 h post-infection, the culture supernatants were assayed for interleukin-1-beta (IL1β) levels using an ELISA kit (eBioscience). Data was expressed as the average IL1β concentration (pg/mL) normalized to CFUs obtained for each strain at 20 h post-infection. Assays were done in three separate experiments (*n* = 3). Statistical analyses were performed using a two-tailed student's *t* test.

### IκBα degradation assays

RAW264.7 cells were infected with wild type SL1344 and the Δ*gogB* strain as described above. At various time points, cells were lysed with PBS/1% Triton X-100 and the lysates were normalized for total protein content. Proteins were resolved by SDS-PAGE and IκBα levels were analyzed by Western blot. Immunoblotting was performed by resolving proteins or cell lysates in 8 or 10% SDS-PAGE and transferring to a PVDF membrane. Blots were analyzed using the following antibodies: rabbit anti-Skp1 (Novus Biologicals), mouse anti-FBXO22 (Abcam), rabbit anti-β-actin (Imgenex), mouse anti-ubiquitin FK2 (Enzo Life Sciences), rabbit anti-GST (Bethyl Laboratories), rat anti-HA-HRP (Roche), rabbit anti-IκBα (Calbiochem), and mouse anti-GAPDH (Novus Biologicals).

### IκBα ubiquitination assay

RAW264.7 cells were seeded in 6 well plates at 2×10^6^ cells/well and infected with wild type SL1344, Δ*gogB*/pgogB, Δ*gogB*/pgogB Δ264–352 and Δ*gogB* strains at an MOI of 20 bacteria per cell in the presence of 10 µM MG-132 (Sigma) to inhibit proteasome degradation of ubiquitinated IκBα. Control wells were infected with the same strains in the presence of 0.2% DMSO. At 30 min post-infection, cells were washed with PBS and fresh media containing 10 µM MG-132 was added to the infected cells and incubated for 3.5 hr. Cells were then lysed with buffer containing 40 mM Tris pH 7.5, 150 mM NaCl, 1% Triton X-100, 1 mM EDTA, 10 µM MG-132, 10 µM N-ethylmaleimide and protease inhibitor cocktail (Roche). Lysates were normalized for protein content and mixed with IgA Dynabeads (Invitrogen) previously coupled to rabbit anti-IκBα following manufacturer's instructions. The mixtures were incubated overnight at 4°C with end-on-end mixing. Beads were then washed three times with TBS/1% Triton X-100 followed by TBS and resuspended in SDS sample buffer. Protein samples were resolved by SDS-PAGE and analyzed by Western blot using HRP-conjugated mouse anti-ubiquitin FK2 (Enzo Life Sciences) and rabbit anti-IκBα (Calbiochem).

### Animal infections and competitive infection assays

Protocols for the infection of experimental animals were approved by the Animal Research Ethics Board at McMaster University and in accordance to guidelines from the Canadian Council on the Use of Laboratory Animals. Competition assays were performed as previously described [45]. Groups of five female C57BL/6 mice were orally inoculated with a mixed inoculum of wild type *Salmonella* and Δ*gogB* resistant to chloramphenicol (∼1×10^6^ CFU per strain). At 72 h post-infection, the bacterial loads were determined by plating tissue homogenates from the liver, spleen, and cecum. The competitive index (CI) was computed as the ratio of mutant/wild type CFU in the output versus input. 129/svImJ mice that are resistant to *Salmonella* due to a homozygous *Nramp^r^* allele were used as experimental animals for chronic *Salmonella* infections. Groups of five female 129/svImJ mice were orally inoculated with 1×10^7^ CFU of either wild type *Salmonella* or the Δ*gogB* strain. After 4, 7, 39, and 60 days of infection, bacterial loads were determined by plating tissue homogenates of the liver, spleen, mesenteric lymph nodes (MLN), and cecum. In some experiments, mice were immunosuppressed prior to infection using dexamethasone in the drinking water as described previously [46]. Data were plotted as geometric means of log-transformed CFU per mg of tissue. Statistical analyses were performed using a nonparametric, two-tailed Mann-Whitney *t* test.

### Histopathological analyses

A portion of the distal cecal tip of experimental animals was fixed in 4% paraformaldehyde (PFA) for 72 h, then transferred to 70% ethanol for 48 h prior to paraffin embedding, sectioning, and hematoxylin-eosin staining. Methodology for histology scoring was performed as previously described [47].

### Real-time PCR (RT-PCR)

*Nramp^r^* mice were orally infected with 10^7^ CFU of wild type and Δ*gogB Salmonella*. At day 4 post-infection, total RNA was extracted from the cecum and MLN using Trizol reagent (Invitrogen) following the manufacturer's protocol. After determining the purity of the extracted RNA, 5 µg of RNA was reverse-transcribed to cDNA using random hexamers and Superscript III reverse transcriptase (Invitrogen) according to manufacturer's protocols. RT-PCR was performed in a Lightcycler 480 (Roche) using the Lightcycler 480 SYBR I Master Mix (Roche) and specific primers listed in Table S2. RT-PCR was performed in triplicate with the following settings: pre-incubation at 95°C for 5 min and 60 cycles of amplification at 95C° for 10 s, 55°C for 10 s, and 72°C for 20 s. Melting curve analysis was done at 95°C for 5 s, 65°C for 1 min and at 97°C followed by cooling to 40°C for 10 s. Data analysis was performed using the Lightcycler 480 version 1.5 software (Roche). Relative fold change in expression was calculated using the formula based on the Pfaffl method: Ratio = [(E_target_)^ΔCt target (control-treated)^]/[(E_ref_)^ΔCt ref (control-treated)^] where E is the primer efficiency determined for specific primers and ΔCt is the difference in the mean crossing thresholds from control uninfected and infected mice. 18S rRNA was used as the reference gene.

## Supporting Information

Figure S1 **Secretion assays of GogB-NT and GogB-CT.** The Δ*gogB Salmonella* containing p*gogB-2HA*, p*gogBNT-2HA* and p*gogBCT-2HA* were grown in SPI-2 inducing media (LPM) at pH 5.8 for 5 h at 37°C. Cells were harvested and the secreted proteins from filtered supernatants were concentrated and resolved by SDS-PAGE and analyzed by Western blot using mouse anti-HA antibodies. Antibodies to DnaK were used as loading control.
(TIF)

Figure S2 **Immunosuppression reduces the bacterial load of the** ***gogB*** **mutant in the cecum.** **A.** Groups of 129/svImJ mice were pretreated with dexamethasone or not, and then orally infected with Δ*gogB* mutant *Salmonella*. At day 4 after infection mice were sacrificed and the bacterial load in the cecum was determined by serial dilution of tissue homogenates. Each data point represents one animal and horizontal bars indicate geometric means. **B.** Groups of 129/svImJ mice were pretreated with dexamethasone and then infected with either wild-type *Salmonella* or the Δ*gogB* mutant. Bacterial load was determined in the cecum at day 4 after infection. Each data point represents one animal and horizontal bars indicate geometric means.
(TIF)

Table S1 **List of plasmids and strains used in this study.**
(DOC)

Table S2 **List of oligonucleotides used in this study.**
(DOC)

## References

[1] Thomson N, Baker S, Pickard D, Fookes M, Anjum M (2004). The role of prophage-like elements in the diversity of *Salmonella enterica* serovars.. J Mol Biol.

[2] Galan JE (1999). Interaction of *Salmonella* with host cells through the centisome 63 type III secretion system.. Curr Opin Microbiol.

[3] Monack DM, Raupach B, Hromockyj AE, Falkow S (1996). *Salmonella typhimurium* invasion induces apoptosis in infected macrophages.. Proc Natl Acad Sci U S A.

[4] Waterman SR, Holden DW (2003). Functions and effectors of the *Salmonella* pathogenicity island 2 type III secretion system.. Cell Microbiol.

[5] Valdez Y, Ferreira RBR, Finlay BB (2009). Molecular mechanisms of Salmonella virulence and host resistance.. Curr Topics Microbiol Immunol.

[6] Wagner PL, Waldor MK (2002). Bacteriophage control of bacterial virulence.. Infect Immun.

[7] Coombes BK, Wickham ME, Brown NF, Lemire S, Bossi L (2005). Genetic and molecular analysis of GogB, a phage-encoded type III-secreted substrate in *Salmonella enterica* serovar typhimurium with autonomous expression from its associated phage.. J Mol Biol.

[8] Quezada CM, Hicks SW, Galan JE, Stebbins CE (2009). A family of *Salmonella* virulence factors functions as a distinct class of autoregulated E3 ubiquitin ligases.. Proc Natl Acad Sci U S A.

[9] Singer AU, Rohde JR, Lam R, Skarina T, Kagan O (2008). Structure of the *Shigella* T3SS effector IpaH defines a new class of E3 ubiquitin ligases.. Nat Struct Mol Biol.

[10] Cardozo T, Pagano M (2004). The SCF ubiquitin ligase: insights into a molecular machine.. Nat Rev Mol Cell Biol.

[11] Kim DW, Lenzen G, Page AL, Legrain P, Sansonetti PJ (2005). The *Shigella flexneri* effector OspG interferes with innate immune responses by targeting ubiquitin-conjugating enzymes.. Proc Natl Acad Sci U S A.

[12] Lomma M, Dervins-Ravault D, Rolando M, Nora T, Newton HJ (2010). The *Legionella pneumophila* F-box protein Lpp2082 (AnkB) modulates ubiquitination of the host protein parvin B and promotes intracellular replication.. Cell Microbiol.

[13] Price CT, Al-Khodor S, Al-Quadan T, Santic M, Habyarimana F (2009). Molecular mimicry by an F-box effector of *Legionella pneumophila* hijacks a conserved polyubiquitination machinery within macrophages and protozoa.. PLoS Pathog.

[14] Sonnberg S, Seet BT, Pawson T, Fleming SB, Mercer AA (2008). Poxvirus ankyrin repeat proteins are a unique class of F-box proteins that associate with cellular SCF1 ubiquitin ligase complexes.. Proc Natl Acad Sci U S A.

[15] Blanie S, Gelfi J, Bertagnoli S, Camus-Bouclainville C (2010). MNF, an ankyrin repeat protein of myxoma virus, is part of a native cellular SCF complex during viral infection.. Virol J.

[16] Kubori T, Galan JE (2003). Temporal regulation of *Salmonella* virulence effector function by proteasome-dependent protein degradation.. Cell.

[17] Patel JC, Hueffer K, Lam TT, Galan JE (2009). Diversification of a *Salmonella* virulence protein function by ubiquitin-dependent differential localization.. Cell.

[18] Marcus SL, Knodler LA, Finlay BB (2002). *Salmonella enterica* serovar Typhimurium effector SigD/SopB is membrane-associated and ubiquitinated inside host cells.. Cell Microbiol.

[19] Winston JT, Strack P, Beer-Romero P, Chu CY, Elledge SJ (1999). The SCFbeta-TRCP-ubiquitin ligase complex associates specifically with phosphorylated destruction motifs in IkappaBalpha and beta-catenin and stimulates IkappaBalpha ubiquitination in vitro.. Genes Dev.

[20] Bai C, Sen P, Hofmann K, Ma L, Goebl M (1996). SKP1 connects cell cycle regulators to the ubiquitin proteolysis machinery through a novel motif, the F-box.. Cell.

[21] Kipreos ET, Pagano M (2000). The F-box protein family.. Genome Biol.

[22] Rohde JR, Breitkreutz A, Chenal A, Sansonetti PJ, Parsot C (2007). Type III secretion effectors of the IpaH family are E3 ubiquitin ligases.. Cell Host Microbe.

[23] Zheng N, Schulman BA, Song L, Miller JJ, Jeffrey PD (2002). Structure of the Cul1-Rbx1-Skp1-F boxSkp2 SCF ubiquitin ligase complex.. Nature.

[24] Samudrala R, Heffron F, McDermott JE (2009). Accurate prediction of secreted substrates and identification of a conserved putative secretion signal for type III secretion systems.. PLoS Pathog.

[25] Angot Al, Peeters N, Lechner E, Vailleau F, Baud C (2006). *Ralstonia solanacearum* requires F-box-like domain-containing type III effectors to promote disease on several host plants.. Proc Natl Acad Sci U S A.

[26] Schulman BA, Carrano AC, Jeffrey PD, Bowen Z, Kinnucan ER (2000). Insights into SCF ubiquitin ligases from the structure of the Skp1-Skp2 complex.. Nature.

[27] Tanaka K, Kawakami T, Tateishi K, Yashiroda H, Chiba T (2001). Control of IkappaBalpha proteolysis by the ubiquitin-proteasome pathway.. Biochimie.

[28] Neish AS, Gewirtz AT, Zeng H, Young AN, Hobert ME (2000). Prokaryotic regulation of epithelial responses by inhibition of IkappaB-alpha ubiquitination.. Science.

[29] Gruenheid S, Gros P (2000). Genetic susceptibility to intracellular infections: Nramp1, macrophage function and divalent cations transport.. Curr Opin Microbiol.

[30] Lawley TD, Chan K, Thompson LJ, Kim CC, Govoni GR (2006). Genome-wide screen for *Salmonella* genes required for long-term systemic infection of the mouse.. PLoS Pathog.

[31] Winter SE, Thiennimitr P, Winter MG, Butler BP, Huseby DL (2010). Gut inflammation provides a respiratory electron acceptor for *Salmonella*.. Nature.

[32] Stecher Br, Robbiani R, Walker AW, Westendorf AM, Barthel M (2007). *Salmonella enterica* serovar typhimurium exploits inflammation to compete with the intestinal microbiota.. PLoS Biol.

[33] Haller D, Holt L, Kim SC, Schwabe RF, Sartor RB (2003). Transforming growth factor-beta 1 inhibits non-pathogenic Gram negative bacteria-induced NF-kappa B recruitment to the interleukin-6 gene promoter in intestinal epithelial cells through modulation of histone acetylation.. J Biol Chem.

[34] Tan MK, Lim HJ, Harper JW (2011). SCFFBXO22 Regulates Histone H3 Lysine 9 and 36 Methylation Levels by Targeting Histone Demethylase KDM4A for Ubiquitin-Mediated Proteasomal Degradation.. Mol Cell Biol.

[35] Van Rechem C, Black JC, Abbas T, Allen A, Rinehart CA (2011). The SKP1-Cul1-F-box and Leucine-rich Repeat Protein 4 (SCF-FbxL4) Ubiquitin Ligase Regulates Lysine Demethylase 4A (KDM4A)/Jumonji Domain-containing 2A (JMJD2A) Protein.. J Biol Chem.

[36] Hicks SW, Charron G, Hang HC, Galan JE (2011). Subcellular targeting of *Salmonella* virulence proteins by host-mediated S-palmitoylation.. Cell Host Microbe.

[37] Knodler LA, Winfree S, Drecktrah D, Ireland R, Steele-Mortimer O (2009). Ubiquitination of the bacterial inositol phosphatase, SopB, regulates its biological activity at the plasma membrane.. Cell Microbiol.

[38] Watson KG, Holden DW (2010). Dynamics of growth and dissemination of *Salmonella* in vivo.. Cell Microbiol.

[39] Hapfelmeier S, Stecher B, Barthel M, Kremer M, Muller AJ (2005). The *Salmonella* pathogenicity island (SPI)-2 and SPI-1 type III secretion systems allow *Salmonella* serovar typhimurium to trigger colitis via MyD88-dependent and MyD88-independent mechanisms.. J Immunol.

[40] Muller AJ, Hoffmann C, Galle M, Van Den Broeke A, Heikenwalder M (2009). The *S*. Typhimurium effector SopE induces caspase-1 activation in stromal cells to initiate gut inflammation.. Cell Host Microbe.

[41] Miao EA, Warren SE (2010). Innate immune detection of bacterial virulence factors via the NLRC4 inflammasome.. J Clin Immunol.

[42] Monack DM, Bouley DM, Falkow S (2004). *Salmonella typhimurium* persists within macrophages in the mesenteric lymph nodes of chronically infected Nramp1+/+ mice and can be reactivated by IFNgamma neutralization.. J Exp Med.

[43] Griffin AJ, Li LX, Voedisch S, Pabst O, McSorley SJ (2011). Dissemination of persistent intestinal bacteria via the mesenteric lymph nodes causes typhoid relapse.. Infect Immun.

[44] Frangioni JV, Neel BG (1993). Solubilization and purification of enzymatically active glutathione S-transferase (pGEX) fusion proteins.. Anal Biochem.

[45] Coombes BK, Lowden MJ, Bishop JL, Wickham ME, Brown NF (2007). SseL is a *Salmonella*-specific translocated effector integrated into the SsrB-controlled *Salmonella* pathogenicity island 2 type III secretion system.. Infect Immun.

[46] Lawley TD, Bouley DM, Hoy YE, Gerke C, Relman DA (2008). Host transmission of *Salmonella enterica* serovar Typhimurium is controlled by virulence factors and indigenous intestinal microbiota.. Infect Immun.

[47] Coburn B, Li Y, Owen D, Vallance BA, Finlay BB (2005). *Salmonella enterica* serovar Typhimurium pathogenicity island 2 is necessary for complete virulence in a mouse model of infectious enterocolitis.. Infect Immun.

