# Supplementary figures and images for: GogB Is an Anti-Inflammatory Effector that Limits Tissue Damage during Salmonella Infection through Interaction with Human FBXO22 and Skp1
Source: PLoS Pathog. 2012 Jun 28;8(6):e1002773. doi: 10.1371/journal.ppat.1002773 (PMC3386239; doi:10.1371/journal.ppat.1002773)

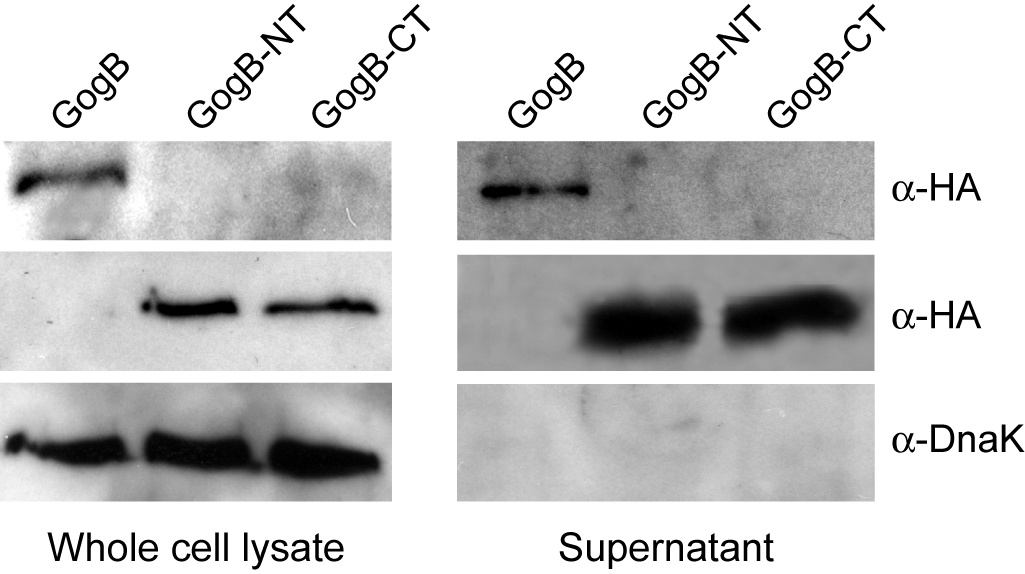

Supplement: Figure S1 — Secretion assays of GogB-NT and GogB-CT. The ΔgogB Salmonella containing pgogB-2HA, pgogBNT-2HA and pgogBCT-2HA were grown in SPI-2 inducing media (LPM) at pH 5.8 for 5 h at 37°C. Cells were harvested and the secreted proteins from filtered supernatants were concentrated and resolved by SDS-PAGE and analyzed by Western blot using mouse anti-HA antibodies. Antibodies to DnaK were used as loading control. (TIF) [file ppat.1002773.s001.tif]

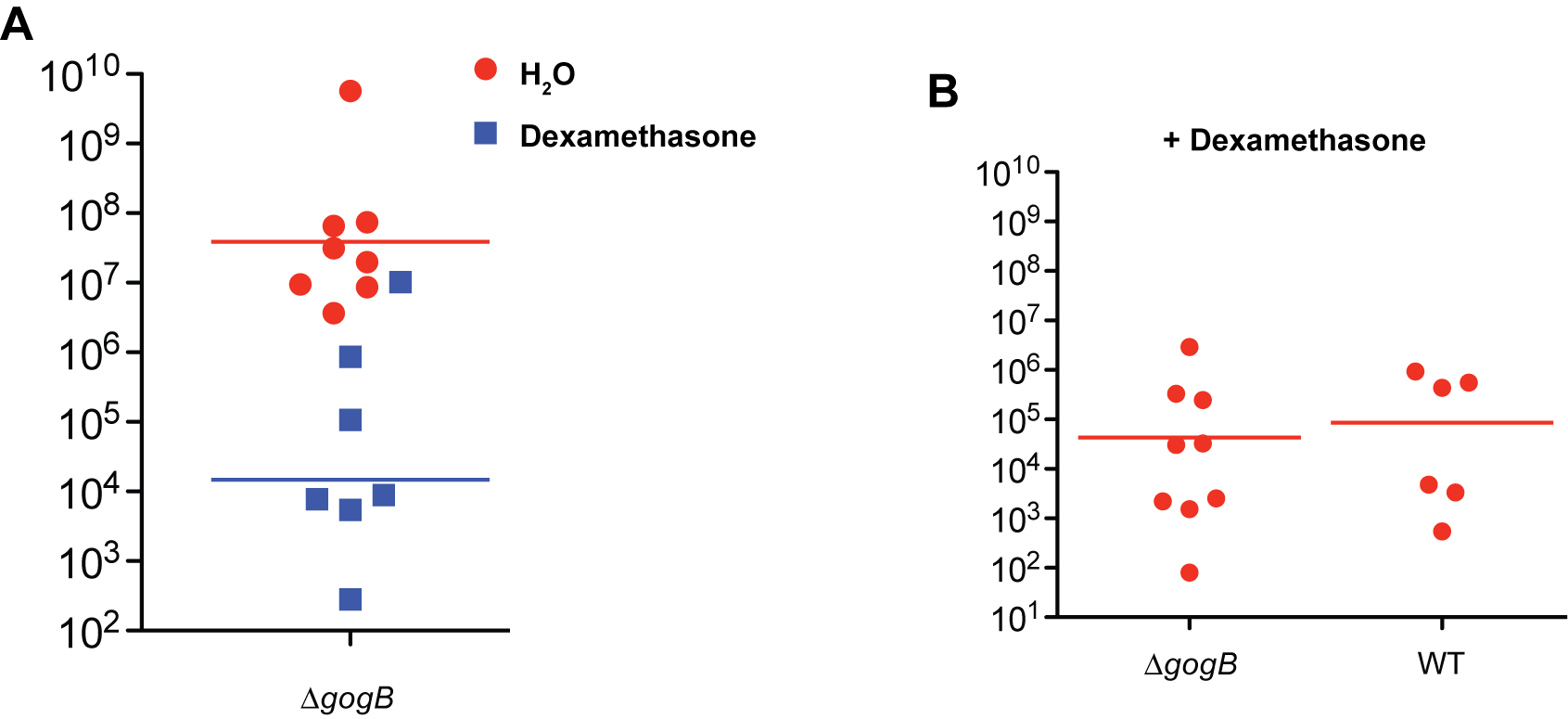

Supplement: Figure S2 — Immunosuppression reduces the bacterial load of the gogB mutant in the cecum. A. Groups of 129/svImJ mice were pretreated with dexamethasone or not, and then orally infected with ΔgogB mutant Salmonella. At day 4 after infection mice were sacrificed and the bacterial load in the cecum was determined by serial dilution of tissue homogenates. Each data point represents one animal and horizontal bars indicate geometric means. B. Groups of 129/svImJ mice were pretreated with dexamethasone and then infected with either wild-type Salmonella or the ΔgogB mutant. Bacterial load was determined in the cecum at day 4 after infection. Each data point represents one animal and horizontal bars indicate geometric means. (TIF) [file ppat.1002773.s002.tif]
