# Supplementary material for: GogB Is an Anti-Inflammatory Effector that Limits Tissue Damage during Salmonella Infection through Interaction with Human FBXO22 and Skp1
Source: PLoS Pathog. 2012 Jun 28;8(6):e1002773. doi: 10.1371/journal.ppat.1002773 (PMC3386239; doi:10.1371/journal.ppat.1002773)
Supplement: Table S1 — List of plasmids and strains used in this study. (DOC) [file ppat.1002773.s003.doc]

**Supporting Information**

Table S1

| **Plasmid and strains** | **Primer sequences** | **Reference** |
| --- | --- | --- |
| pWSK129 *gogB-2HA* |  | Coombes, 2005 |
| pW­SK129 *gogBNT_1-253_-2HA* | F – acgcgtcgacgtcaataatcagcgcct  R – gccgagatctgatagactttaaattaacaggaagc | This study |
| pWSK129 *gogBCT_254-497_-2HA* | F – actagtcgacgtcaataatcagcgcctgcagcg  R1 – aggggtccttgaggataaattgtttgatgtcgcctcgttact  R2 – ggaagatctacgatttctatttttaggcttatatttatcc | This study |
| pWSK129 *gogB* Δ*264-352-2HA*  (ΔFbox) | F1 – actagtcgacgtcaataatcagcgcctgcagcg  R1 – aaacacggctctattcttccatgcaataggggtcct  F2 – aagaatagagccgtgtttaataaagatgagaag  R2 – ggaagatctacgatttctatttttaggcttatatttatcc | This study |
| pWSK129 *gogB-L^270^P^271^/AA-2HA* | F – GGAAATACCAACCTGCAATGCCGCTGCACATATAGATATTAG  R - CTAATATCTATATGTGCAGCGGCATTGCAGGTTGGTATTTCC | This study |
| pWSK129 *gogBCT2_254-497_-L^47^P^48^/AA-2HA* | F – GGAAATACCAACCTGCAATGCCGCTGCACATATAGATATTAG  R - CTAATATCTATATGTGCAGCGGCATTGCAGGTTGGTATTTCC | This study |
| pGEX*-6P-1-gogB* | F – ggcgggatccatgacatatagattg  R – ccgtgtcgactcaacgatttctattttttagg | This study |
| pGEX*-6P-1-gogBNT_1-253_* | F – ggcgggatccatgacatatagattg  R – gcctgtcgactcagatagactttaaattaac | This study |
| pGEX*-6P-1-gogBCT_253-497_* | F – ggtgggatccatgaatttatcctcaagg  R – ccgtgtcgactcaacgatttctattttttagg | This study |
| SL1344 *gogB::Cm^r^* |  | Coombes, 2005 |
| SL1344 *gogB::Cm^r^ sspH2::Kan^r^* |  | This study |
