# Supplementary material for: GogB Is an Anti-Inflammatory Effector that Limits Tissue Damage during Salmonella Infection through Interaction with Human FBXO22 and Skp1
Source: PLoS Pathog. 2012 Jun 28;8(6):e1002773. doi: 10.1371/journal.ppat.1002773 (PMC3386239; doi:10.1371/journal.ppat.1002773)
Supplement: Table S2 — List of oligonucleotides used in this study. (DOC) [file ppat.1002773.s004.doc]

**Supporting Information**

**Table S2**

| **Primer** | **Primer sequence** |
| --- | --- |
| TNFα | F – TCTTCTCATTCCTGCTTGTGG  R – GGTCTGGGCCATAGAACTGA |
| IL1β | F – TGTAATGAAAGACGGCACACC  R – TCTTCTTTGGGTATTGCTTGG |
| IL4 | F – GAGAGATCATCGGCATTTTGA  R – AGCCCTACAGACGAGCTCAC |
| IL10 | F – CAGAGCCACATGCTCCTAGA  R – GTCCAGCTGGTCCTTTGTTT |
| IL12p40 | F – atcgttttgctggtgtctcc  R – ggagtccagtccacctctaca |
| MIP2 | F- AAAATCATCCAAAAGATACTGA  R – CTTTGGTTCTTCCGTTGAGG |
| TGFβ1 | F – TGGAGCAACATGTGGAACTC  R – CAGCAGCCGGTTACCAAG |
